# Extensive tracheal resection in lung cancer and tuberculosis: a case report

**DOI:** 10.1186/s12890-020-01230-7

**Published:** 2020-07-18

**Authors:** Dmitry Giller, Boris Giller, Galina Scherbakova, Elizaveta V. Mikhaylenko, Liudmila M. Mikhaleva, Vladimir N. Nikolenko, Liliya V. Gavryushova, Siva G. Somasundaram, Cecil E. Kirkland, Gjumrakch Aliev

**Affiliations:** 1 M.I. Perelman Department of Phthisiopulmonology and Thoracic Surgery, I.M. Sechenov First Moscow State Medical University (Sechenov University), Moscow, Russian Federation; 2 Department of Human Anatomy, N. V. Sklifosovsky Institute of Clinical Medicine, I. M. Sechenov First Moscow State Medical University (Sechenov University), Moscow, Russian Federation; 3 Laboratory of Cellular Pathology, Research Institute of Human Morphology, 3 Tsyurupy Street, Moscow, Russian Federation 117418; 4 Department of Normal and Topographic Anatomy, M.V. Lomonosov Moscow State University, Leninskie Gory, 1, Moscow, 119991 Russia; 5 Department of Therapeutic Dentistry, Saratov State Medical University named after V.I. Razumovsky, 410012 Saratov, Russia; 6 Department of Biological Sciences, Salem University, Salem, WV 26426 USA; 7 Institute of Physiologically Active Compounds of Russian Academy of Sciences, 1 Severny pr., Chernogolovka, Moscow Region, 142432 Russia; 8 GALLY International Research Institute, 7733 Louis Pasteur Drive, #330, San Antonio, TX 78229 USA

**Keywords:** Airway obstruction, Bronchoconstriction, Lung cancer, Tracheal bifurcation resection, Tuberculosis

## Abstract

**Background:**

Tracheal bifurcation resection remains the greatest challenge in airway reconstruction, especially with extensive lesions. Additionally, lung cancer and pulmonary tuberculosis comorbidity complicate the chemoradiotherapy treatment due to the TB reactivation. This case describes tracheal resection in a patient with both tuberculosis (TB) and lung cancer.

**Case presentation:**

The patient was diagnosed with right lung tuberculosis and upper lobe cancer with trachea invasion complicated by hemoptysis. A right pneumonectomy with circular trachea bifurcation resection was performed. Radiotherapy and chemotherapy were not administered to avoid TB reactivation. At 5.5 years post-surgery, there was cancer recurrence that was treated with radiation therapy. At 10 years post-surgery, an invasive squamous-cell carcinoma of a three-segment bronchus on the left was revealed. Radiation therapy and a course of chemotherapy were carried out with almost complete tumor regression.

**Conclusions:**

TB presence should not serve as a basis for the refusal of cancer treatment. Combined treatment may be recommended when the main infection focus in the pulmonary parenchyma is removed during surgery.

## Background

Tracheal bifurcation resection remains the greatest challenge in airway reconstruction, especially with extensive lesions. Some authors have stated that resection exceeding 3–5 cm or 4–5 tracheal half-rings is impossible [1, 2]. Other research has indicated that chemoradiotherapy for lung cancer in the presence of tuberculosis (TB) is not advised due to possible complications from simultaneous therapy [3, 4]. This clinical case presents a description of an extensive tracheal resection in a patient with both lung cancer and TB.

## Case presentation

A 53-year-old male, a smoker (32 packs/year) was admitted to the Central Research Institute of Tuberculosis (CRIT), Moscow, Russia in 2008. He presented complaints of general weakness, cough with irregular mucopurulent sputum, fever up to 37.8 °C, and chest discomfort. The patient had neither family nor work contact indicating exposure to TB. The lungs had small bubbling rales in the upper sections on the right.

From the anamnesis, it is known that in December 2005 upper-lobar pneumonia on the right was diagnosed. Wide-spectrum antibiotics were prescribed with a positive effect. A fibrobronchoscopy was not performed.

**Fig. 1:**
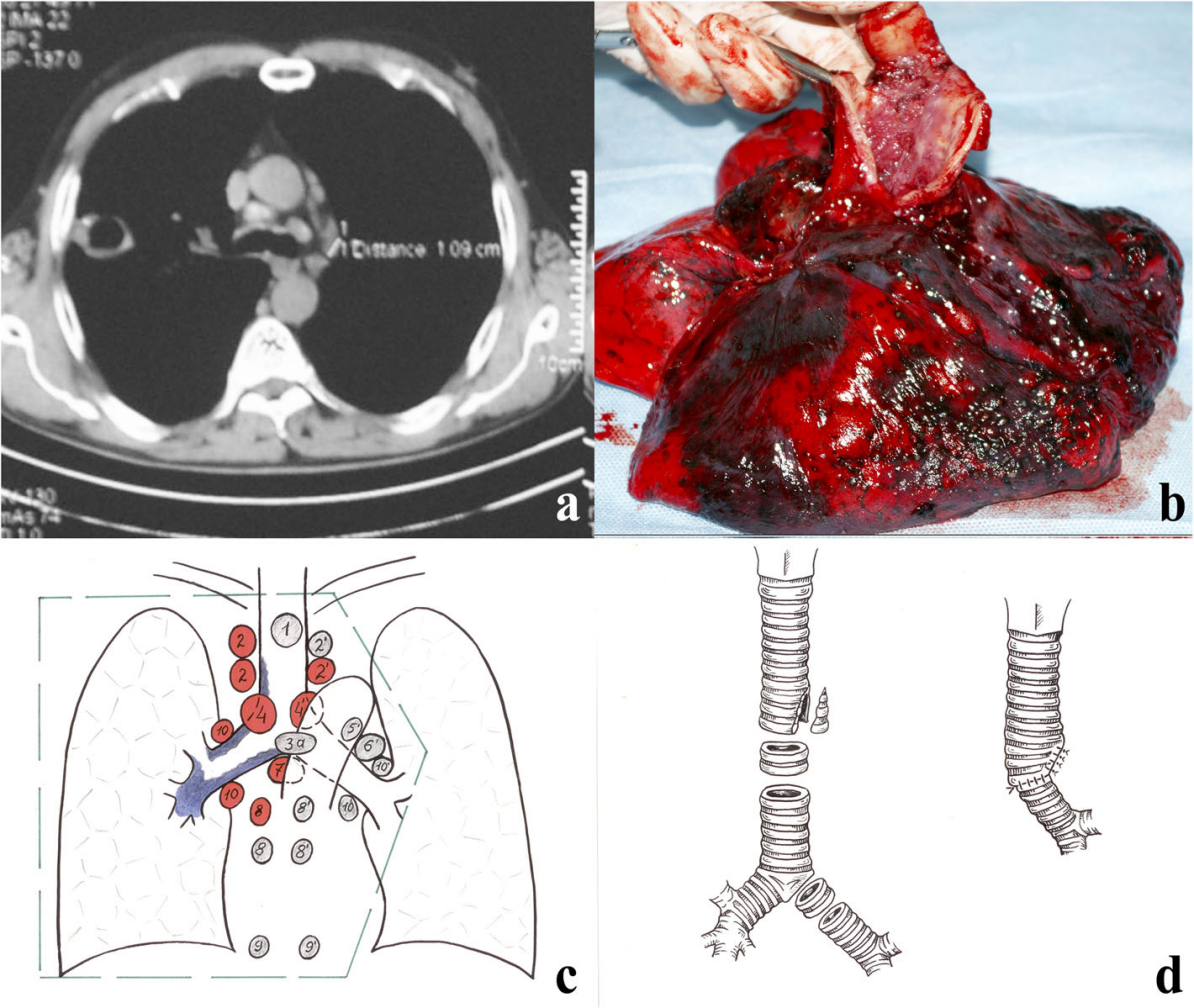
Clinical and imaging manifestation of the case. **a** CT scan prior to surgery (4.0 cm cavity in the right upper lobe with focal seeding; mediastinal lymph nodes enlargement); **b** Lung gross section; **c** Lymphadenectomy volume and metastasis spread; **d** Tracheobronchial reconstruction scheme

In 2007, cavitary tuberculosis of the right upper lobe was diagnosed (Fig. 1a). MTB resistant to isoniazid and rifampicin was found in sputum. Fibrobronchoscopy has revealed cancer of the right main bronchus with a transition to the lower third of the trachea and the close sections of the left main bronchus. Highly differentiated squamous cell carcinoma (SCC) was cytologically and histologically verified.

Spirometric parameters — vital capacity and forced expiratory volume in first second amounted to 105.8 and 62.4%, respectively; the partial pressures of blood gases were pO2–67 mmHg, pCO2–35 mmHg; the 6-min walking test was 480 m. Six months of anti-tubercular treatment in the TB dispensary were unsuccessful. A fibrous cavity in the left upper lobe and bacterial excretion in the sputum remained. The patient was examined at oncological institutions in Moscow and Yaroslavl. The tumor was identified as unresectable, and chemoradiotherapy were determined to be inappropriate due to the TB presence.

In 2008, the patient was referred to the CRIT. Based on the sputum cultural results and X-ray pictures, fibro-cavitary TB of the right lung was diagnosed (Fig. 1b). Central peribronchial highly-differentiated SCC of the right upper lobe with trachea invasion, T4N3M0 (IIIB), complicated by hemoptysis, was diagnosed based on histological and cytological studies and X-rays. Due to the specific diagnostic methods’ applications, there was no doubt about the diagnosis. Given the presence of TB and the possibility of life-threatening pulmonary hemorrhage in a patient who, otherwise, was in a relatively good condition, we determined that surgical treatment was practicable, despite lung cancer spread on the background of anti-TB therapy (ofloxacin, amikacinum, prothionamide, ethambutol, pyrazinamide).

**Fig. 2:**
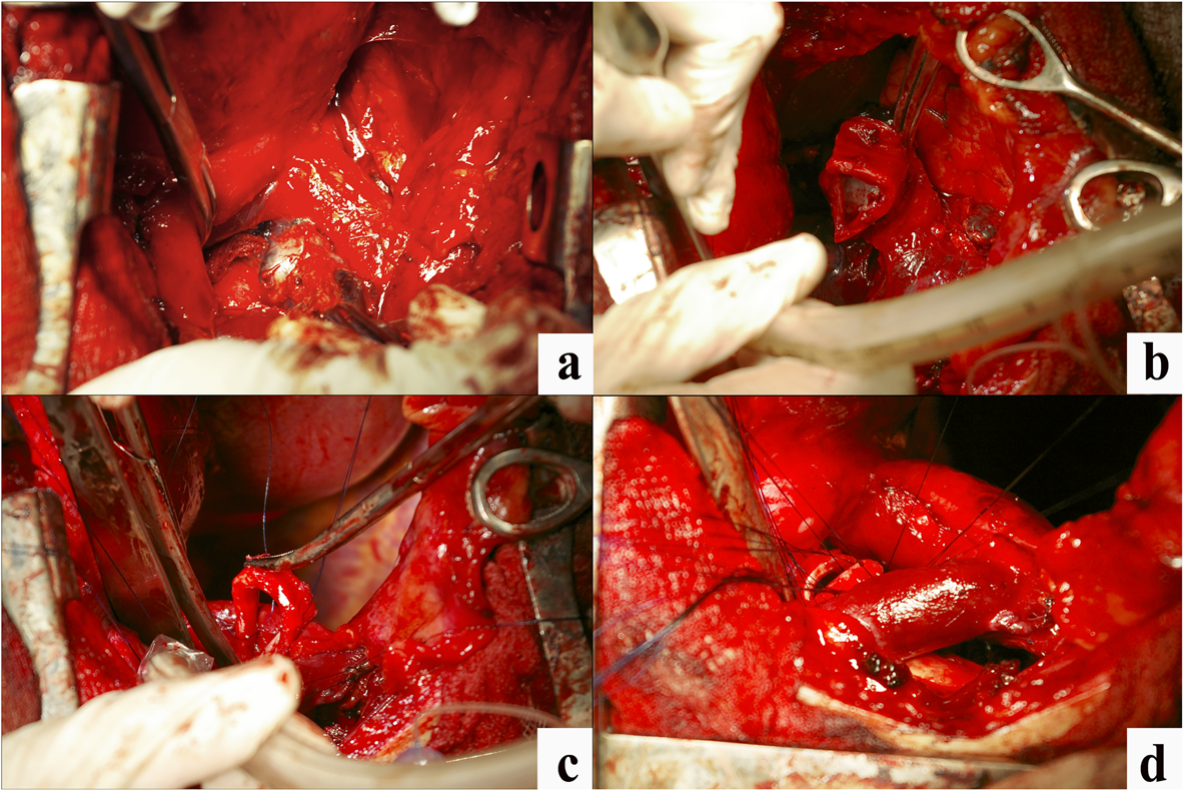
Bilateral lymphadenectomy along with tracheal bifurcation circular resection in a patient with lung cancer and TB; **a** Bifurcation lymph nodes dissection; **b** Trachea bifurcation circular excision; **c** Additional trachea excision; **d** Tracheobronchial anastomosis

An extended combined right pneumonectomy was performed with circular resection of the tracheal bifurcation and bilateral lymphadenectomy from transsternal access using combined endotracheal anesthesia (Fig. 2a-d). A bilateral mediastinal dissection was performed from the median sternotomy with the excision of 1, 2, 3a, 4–9 groups of lymph nodes (Fig. 1c). The right lung with a tracheal segment and one left main bronchus half-ring were removed together with the palpation borders. At this stage, a left main bronchus jet-ventilation was performed through the sternotomy.

After evaluating the possibility of further resection, two more tracheal and two left main bronchus half-rings were dissected. The final resection line of the trachea and bronchus was 0.5–1.0 cm from the tumor’s edge. An urgent histological examination of the resection line was not carried out because a larger volume of resection was considered impossible. The diastasis between the trachea and the left main bronchus after complete excision of the pathology was more than 5 cm. Wedge resection of the tracheal cartilage portion on the left border with a membranous part was followed by defect suturing. High-tension anastomosis formation on the jet ventilation tube was performed to narrow the tracheal diameter to that of the bronchial stump (Fig. 1d).

The patient’s chin was fixed to his chest with one suture for 3 weeks. The surgery duration was 330 min, and intraoperative blood loss was 200 ml. A blood transfusion was not performed. The postoperative period was uncomplicated.

Histological, cytological, and microbiological studies of the surgical material confirmed TB and SCC of the trachea and right upper lobe bronchus combination. No tumor invasion was detected along the resection line of the trachea and bronchus.

On the 21st day after the surgery, the patient was discharged in satisfactory condition and routed to an oncology dispensary at his place of residence. Radiochemotherapy cancer therapies were not used due to fears of TB reactivation. The patient received anti-TB drugs according to the preoperative treatment regimen for 12 months.

Four months post-surgery, the patient was admitted again with stridor. A fibrobronchoscopy revealed granulation stenosis of the anastomosis. Under intravenous anesthesia, the stenosis area was stented. The stent was removed after 14 months with airway restoration.

With subsequent follow-up, there was no TB relapse. In 2014 (5.5 years post-surgery), cancer recurrence in the upper third of the trachea along the anastomosis line was diagnosed. Radiation therapy was performed using a Theratron, resulting in complete tumor regression.

**Fig. 3:**
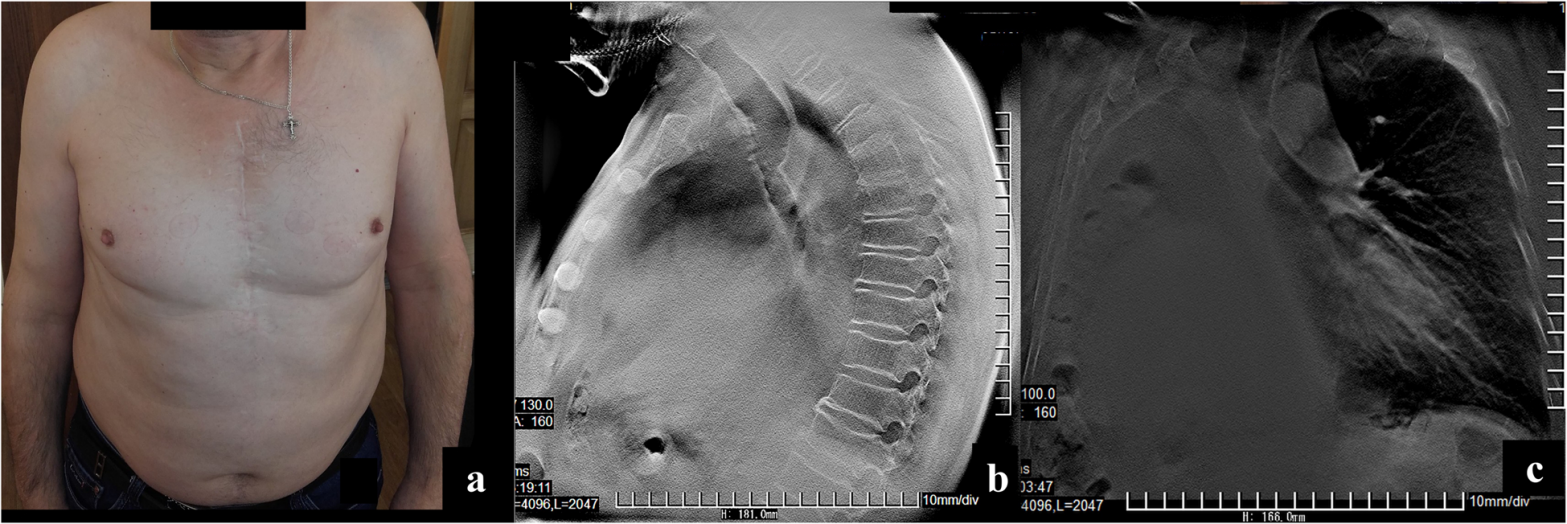
The results of combined radiation therapy and chemotherapy 10 years after surgery; **a** Patient 10 years post-surgery; **b**&**c** Tomosynthesis 10 years post-surgery

**Fig. 4:**
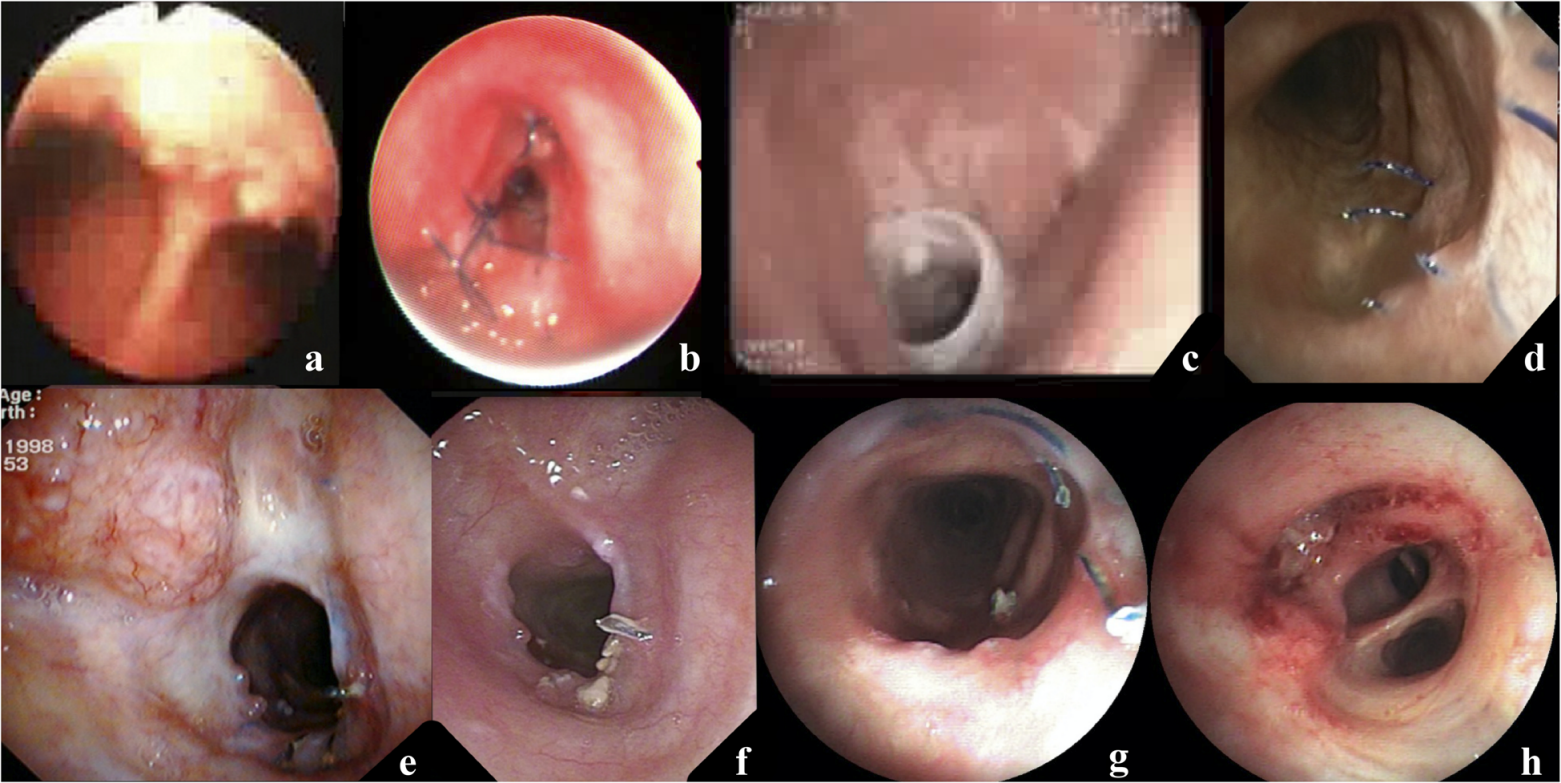
**a** The tracheal bifurcation of patient before surgery; **b** Granulation stenosis of the anastomosis, 4 months past surgery; **c** The stent is installed after tracheobronchial anastomosis dilatation; **d** The tracheobronchial anastomosis zone, 5 years past surgery; **e** Cancer relapse in the trachea above the anastomosis, 5 years 5 months past surgery; **f** The anastomosis, 7 years past surgery. Complete tumor regression after radiation therapy; **g** The anastomosis, 9 years past surgery with free passage; **h** Endoscopy, 9 years and 10 months past surgery. The orifice of the upper zone bronchus of a single left lung is completely obstructed by tumor tissue

In April 2018, a central cancer of a three-segment bronchus on the left was revealed (an invasive highly differentiated squamous non-keratinized carcinoma). Radiation therapy and a course of chemotherapy (20 mg of nambin) were carried out with almost complete tumor regression. In July 2018 (10 years after the surgery), the patient’s condition is satisfactory and he reported no similar symptoms. (Fig. 3a-c, Fig. 4a-h).

## Discussion and conclusions

Oncologists had rejected chemoradiation therapy application in our patient due to TB, and surgery was performed due to the likelihood of pulmonary hemorrhage. In Russia and some post-Soviet Union countries, active TB is considered a contraindication for radiochemotherapy [3]. However, there are studies that report the combination of antitubercular with anticancer chemotherapy and radiotherapy does not increase the number of complications or negative outcomes of treatment [5, 6]. Postoperative radiotherapy could possibly prevent local recurrence in this case.

Despite the large number of available techniques to mobilize the airway for extensive trachea resections, we found few studies on successful resection of over 50% of the trachea [7, 8]. The majority of them were performed with the left main bronchus amputation from the bifurcation and its anastomosis with intermediate bronchus or with the left lung removal, or with leaving it in atelectasis.

Completely different conditions are presented during right pneumonectomy with extensive resection of trachea bifurcation and its chest part. When there is a risk of bending and anastomosis zone compression under the aortic arch exists, this severely limits the possibility of expanding the resection zone.

**Fig. 5:**
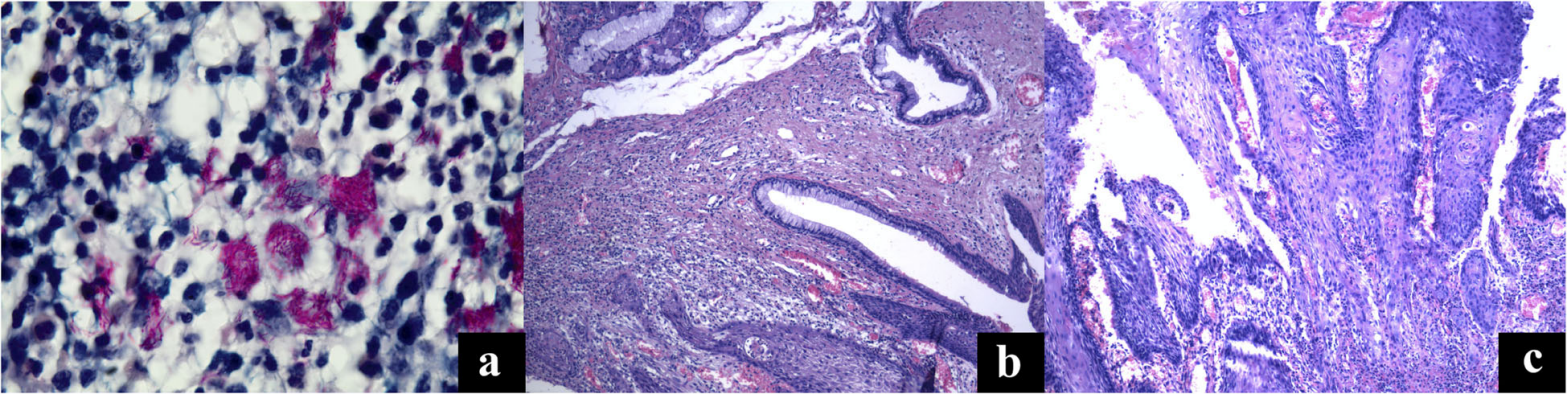
Histological *microphotographs*; **a***Mycobacterium tuberculosis* in the area of active tuberculosis inflammation in the bronchial wall. × 1000. Ziehl-Nielsen coloring; **b** The transition zone of dysplastic bronchial epithelium into squamous cell cancer in the area of tuberculous inflammation. × 200. Hematoxylin and eosin stain; **c** SCC of the cartilage bronchus. X200. Hematoxylin and eosin stain

In our case, a broad resection of the trachea thoracic part and bifurcation was done. Despite complete mobilization of the remaining lung hilum elements, the strong tension has a tendency to anastomotic granulation stenosis. An additional risk factor for granulation stenosis (Fig. 5a-c) was active bronchial tuberculosis, detected in the resected part.

The presence of TB should not serve as a basis for combined lung cancer treatment. Combined treatment may be recommended when the main infection focus in the pulmonary parenchyma is removed during surgery.

## Data Availability

All data are available in the manuscript [and its supplementary information files].

## Abbreviations

TB: Tuberculosis
CRIT: Central Research Institute of Tuberculosis
SCC: Squamous cell carcinoma
